# Leveraging electronic data to expand infection detection beyond traditional settings and definitions (Part II/III)

**DOI:** 10.1017/ash.2022.342

**Published:** 2023-02-10

**Authors:** Westyn Branch-Elliman, Alexander J. Sundermann, Jenna Wiens, Erica S. Shenoy

**Affiliations:** 1 Section of Infectious Diseases, Department of Medicine, Veterans’ Affairs (VA) Boston Healthcare System, Boston, Massachusetts; 2 VA Boston Center for Healthcare Organization and Implementation Research (CHOIR), Boston, Massachusetts; 3 Harvard Medical School, Boston, Massachusetts; 4 Division of Infectious Diseases, Department of Medicine, University of Pittsburgh, Pittsburgh, Pennsylvania; 5 Division of Computer Science and Engineering, University of Michigan, Ann Arbor, Michigan; 6 Infection Control Unit, Massachusetts General Hospital, Boston, Massachusetts; 7 Division of Infectious Diseases, Department of Medicine, Massachusetts General Hospital, Boston, Massachusetts

## Abstract

The rich and complex electronic health record presents promise for expanding infection detection beyond currently covered settings of care. Here, we review the “how to” of leveraging electronic data sources to expand surveillance to settings of care and infections that have not been the traditional purview of the National Healthcare Safety Network (NHSN), including a discussion of creation of objective and reproducible infection surveillance definitions. In pursuit of a ‘fully automated’ system, we also examine the promises and pitfalls of leveraging unstructured, free-text data to support infection prevention activities and emerging technological advances that will likely affect the practice of automated infection surveillance. Finally, barriers to achieving a completely ‘automated’ infection detection system and challenges with intra- and interfacility reliability and missing data are discussed.

The power of surveillance as a tool to prevent healthcare-associated infections (HAIs) is that it can be used to identify quality improvement opportunities and then to monitor the impact of prevention activities over time. Traditionally, most surveillance activities have focused on a relatively narrow segment of clinical care—inpatient settings and some procedural care areas. Inpatient care represents a particularly high-risk period for HAIs due to the high proportion of patients with indwelling devices that increase risk, and due to complex and high-risk procedures.

The continued movement of care outside of inpatient settings^1^ creates a need for evolving infection prevention strategies and surveillance activities and a need to develop novel strategies to expand infection prevention efforts beyond the inpatient world in an efficient and sustainable way. Electronic algorithms applied to electronic health records (EHRs) are a promising means of achieving such expansion. Emerging technologies also offer the promise of a ‘fully automated’ infection surveillance system that uses EHR data to accurately measure infections theoretically without the need for human resources or manual review.

Currently, the Centers for Disease Control and Prevention’s (CDC) National Healthcare Safety Network (NHSN) surveillance activities focus primarily on inpatient settings and on a relatively narrow spectrum of HAIs. Objective NHSN definitions are designed to allow for interfacility comparisons, and are, in turn, used to inform hospital quality rankings and Centers for Medicare and Medicaid Services (CMS) reimbursements as part of the Hospital Compare Program. In a companion review, we have discussed current realities of automated strategies for facilitating HAI surveillance.^2^

In this article, we focus the ways current technology and electronic data are used to expand HAI surveillance activities to include non-mandated, but clinically important, healthcare-associated infections and settings. Challenges created by electronic data collection are highlighted, including how these barriers limit the potential for a ‘fully automated’ infection detection system. In an accompanying review, we discuss how these electronic tools can be combined with technological and genomics innovations to revolutionize the practice of infection prevention and to achieve a true ‘learning healthcare system,’ in which data are analyzed in near real time and are translated into actions that improve bedside clinical care.^3^

## The continued movement from inpatient to outpatient and invasive to minimally or noninvasive care

Although inpatient settings are inherently enhanced for higher probability HAI cases, most clinical care is provided in outpatient settings. Additionally, surgeries that previously took place in inpatient settings, such as total knee replacements and hysterectomies, are increasingly managed as outpatient procedures. Furthermore, traditional surgical procedures are being replaced by less invasive procedures performed in outpatient settings or by other specialties, such as interventional radiology. These outpatient surgeries and less invasive procedures on an individual basis generally present lower risk of HAI; however, in aggregate due to larger patient volume, they may contribute substantially to the overall HAI burden.^4,5^

## Expanding surveillance activities: Do not let the perfect be the enemy of the better

For *mandated* HAI surveillance, accuracy is essential for several reasons, such as identifying areas of potential improvement and focusing scarce infection prevention resources on areas that will benefit the most. Measurements can also affect the hospital’s reimbursements, hospital comparisons, and ultimately facility reputation. For *non-mandated* surveillance, goals and expectations are different. Entities that expand infection detection activities need to balance feasibility with improvement and innovation. In clinical areas with no surveillance and limited quality improvement support, incremental expansion and identification of sentinel events can substantially improve care delivery—even if not every case is counted and identified and the denominator is not perfectly calibrated. As such, they are ideal targets for electronically augmented infection surveillance efforts.

A major challenge for expansion into non-inpatient settings is the inherent difficulty in identifying extremely rare events. For example, let us assume that the rate of surgical-site infection (SSI) is 1% following traditional surgeries and 0.1% following less invasive but increasingly frequent interventional radiology procedures. Next, we consider an EHR-based tool developed to flag true infection cases for chart review and more in-depth data collection. Assuming a sensitivity of 95% and a specificity of 95%—both quite high—the positive predictive value (PPV) of the SSI detection tool is 16%, translating to 6.2 charts requiring manual review for every true infection identified. When the same operating characteristics are applied to the lower-prevalence event, the positive predictive value falls to 1.8%, or manual review of 54 charts to identify 1 true infection. Thus, an EHR-based tool with the same test characteristics is inherently less operationally useful as the event rate decreases.^6^ Given the current workforce challenges facing infection prevention and control, strategies that spare human resources are essential. Thus, when expanding infection surveillance to evaluate low-frequency but high-volume areas, focusing on strategies to maximize electronic-algorithm PPV may balance case ascertainment with personnel resources required to identify true cases.

## Electronic data elements and tools to support automated infection detection

Due to high case volume, relative rarity of events, and the more diffuse nature of the clinical care that is provided outside of the inpatient settings, novel tools and strategies are needed to expand infection surveillance beyond what is currently required. With multidisciplinary collaboration and substantial resource allocation, automated infection detection methods present a path forward.

The cornerstone of automated infection surveillance has been structured data elements such as laboratory results, vital sign measurements, or other data elements that are collected in a defined and organized format within the EHR (Table 1). These structured data elements are relatively straightforward to extract and are amenable to the application of logical statements; they have been described in detail in the first review in this series.^2^ Unstructured data, such as clinical documentation, is also a rich source of information in the EHR but is less amenable to automation given the specifics of how data are collected.

**Table 1.** Potentially Automated Electronic Data Elements and Relative Complexity of Automation

Relative Complexity of Automation	Variable
**Low to moderate (structured data elements)**	
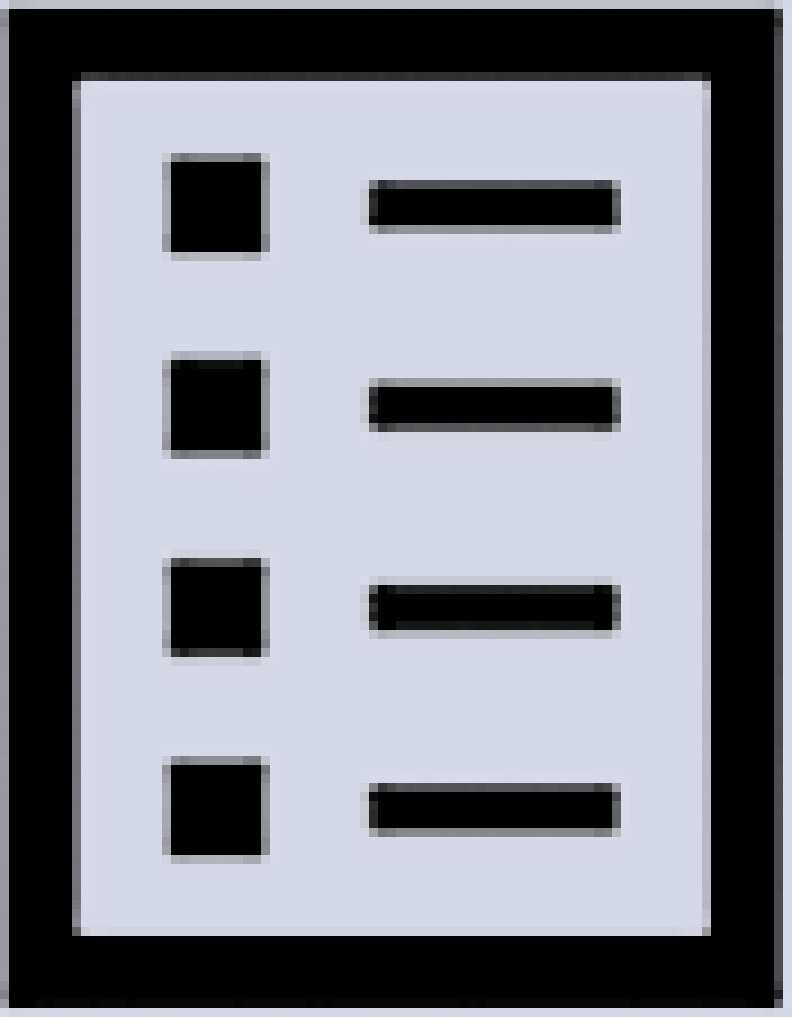	CPT codes/denominator assessments
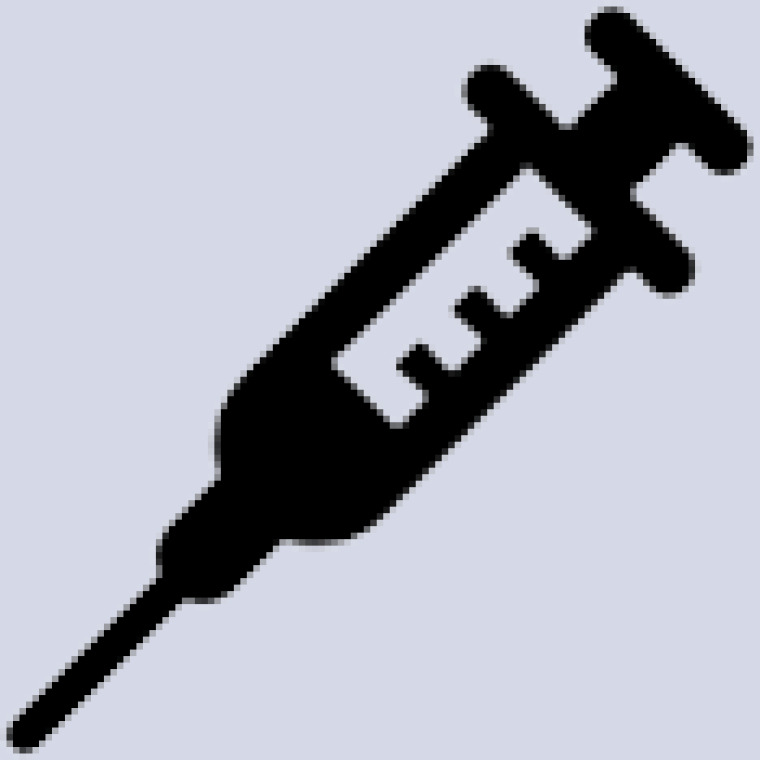	Incision and drainage procedures, repeat procedures
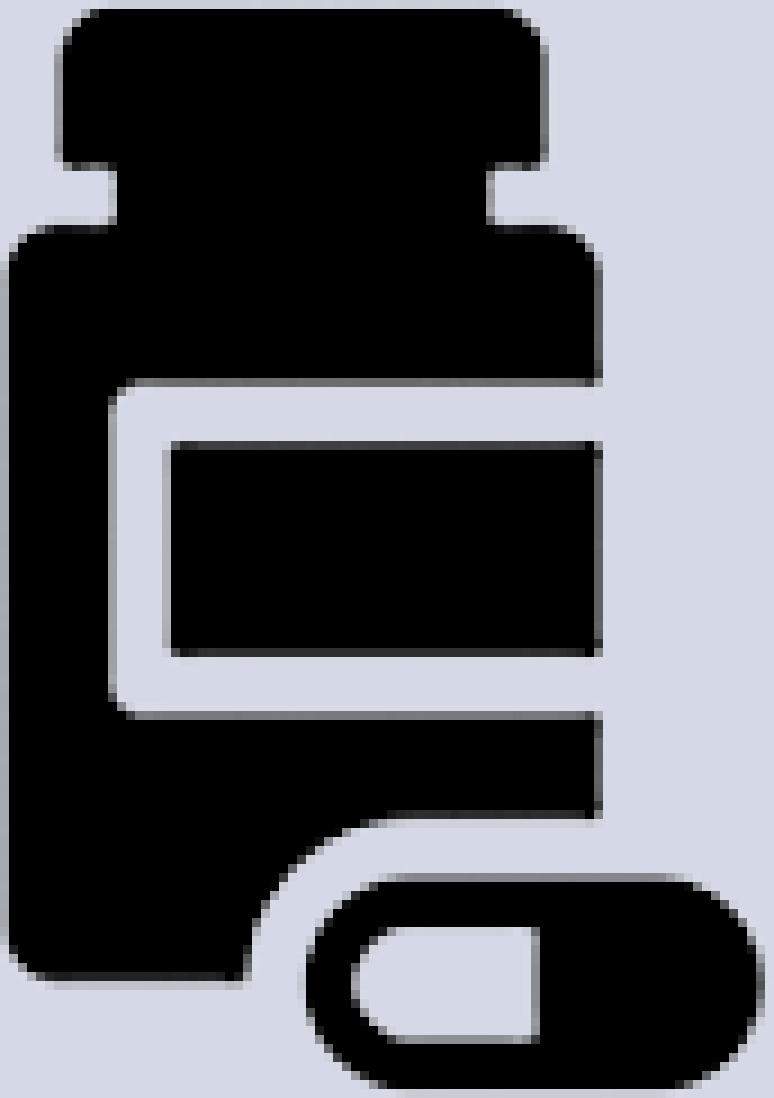	Antimicrobial orders and prescriptions
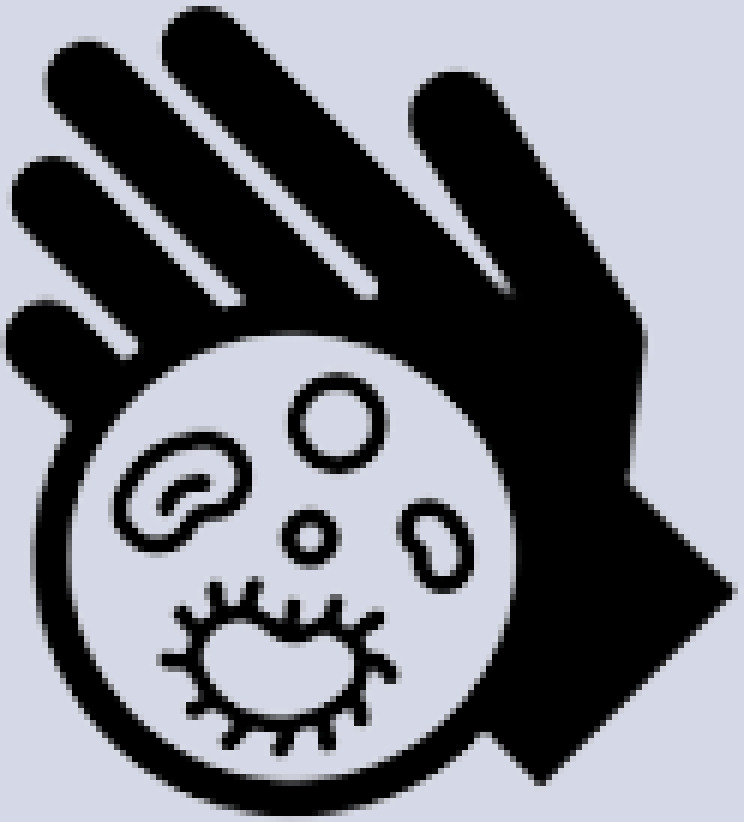	Microbiology orders
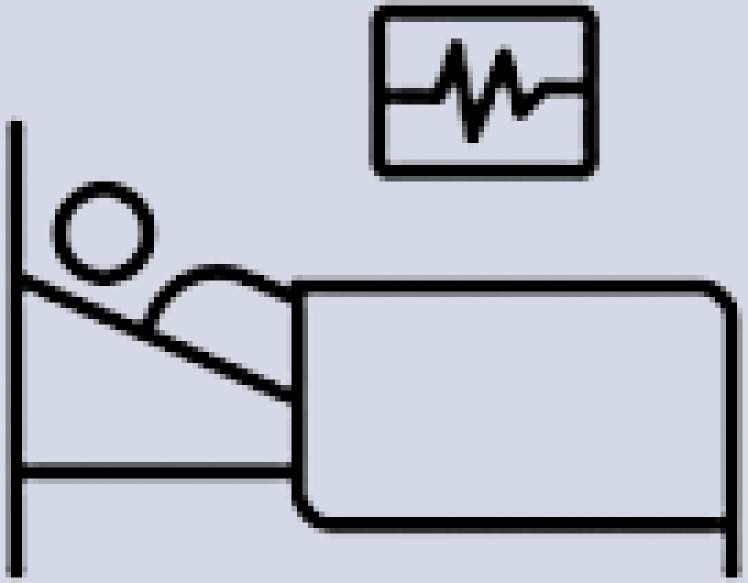	Emergency room visits, hospital admissions, repeat procedures
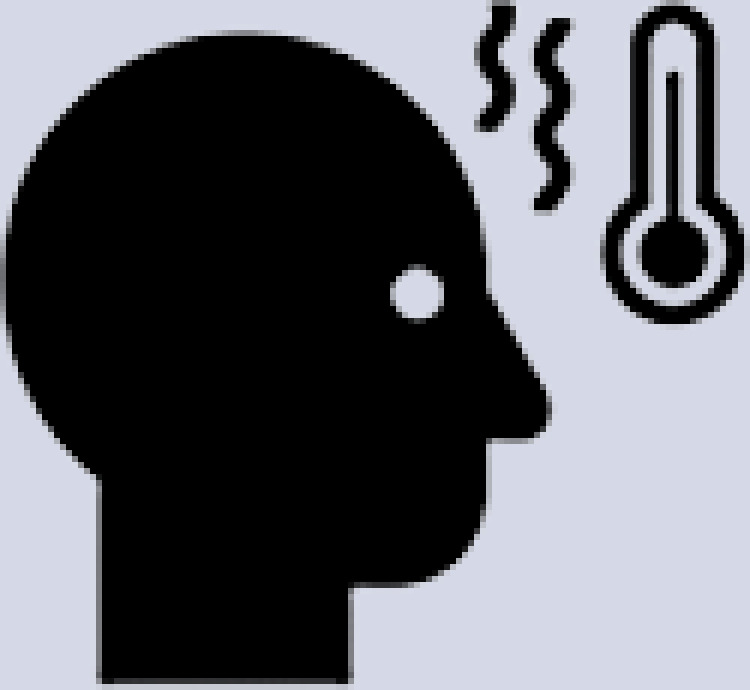	Vital signs
**Moderate to high (unstructured data elements, data from images and genomics)**	
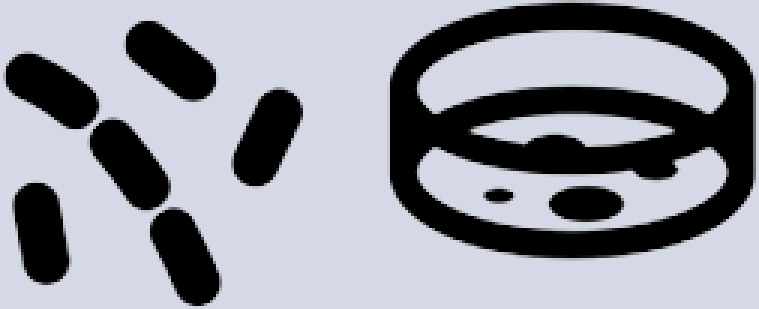	Microbiology and other clinical results
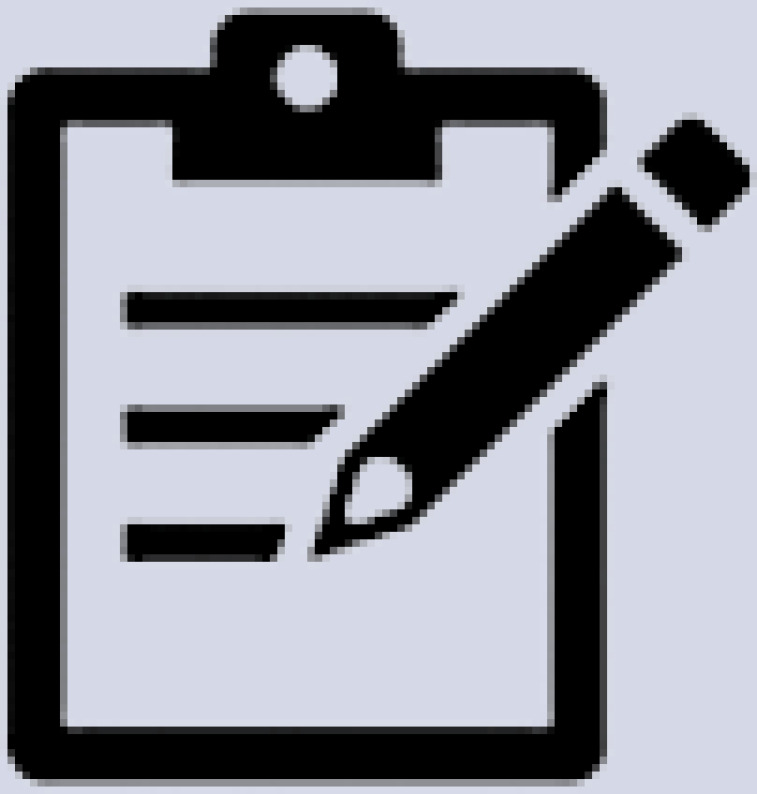	Clinical notes and discharge summaries
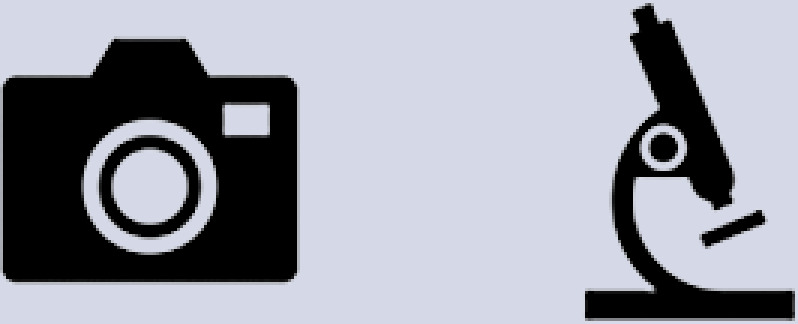	Imaging and pathology reports, information extracted directly from images
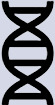	Genomics

Note. CPT, current procedural terminology.

## Leveraging electronic health records and tools to expand surveillance: Who, what, where, why, and how?

### Developing working infection surveillance definitions: Defining “ground truth” to train automated tools

Expanding infection prevention beyond the NHSN requires adaptation or creation of surveillance definitions that can be systematically and reliably applied to measure infections (Table 2). For example, one might want to expand methicillin-resistant *Staphylococcus aureus* (MRSA) infection surveillance to outpatient dermatology visits and biopsies. When expanding surveillance activities, the first questions to ask are these: What is the highest priority of the organization and are there specific areas or settings where quality improvement is needed or expected? Is there a request from a specific provider based on a few identified infections that may signal a need for intervention? Did a sentinel event occur? Answers to these sorts of questions dictate the next steps.

**Table 2.** Examples of Infection Surveillance Expansion Opportunities and Potential Sources of Definitions

Category Listed In Decreasing Complexity	Clinical Example(s)	Possible Sources of Definitions
Adapting/Expanding to new setting or procedure	Postarthroscopy surgical-site infections	NHSN surgical-site infection definitions for hip or knee replacement procedures
	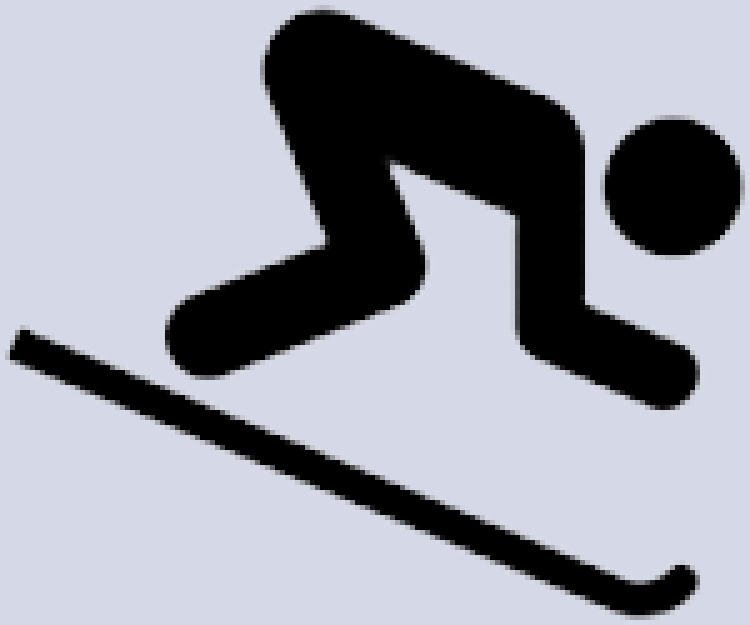	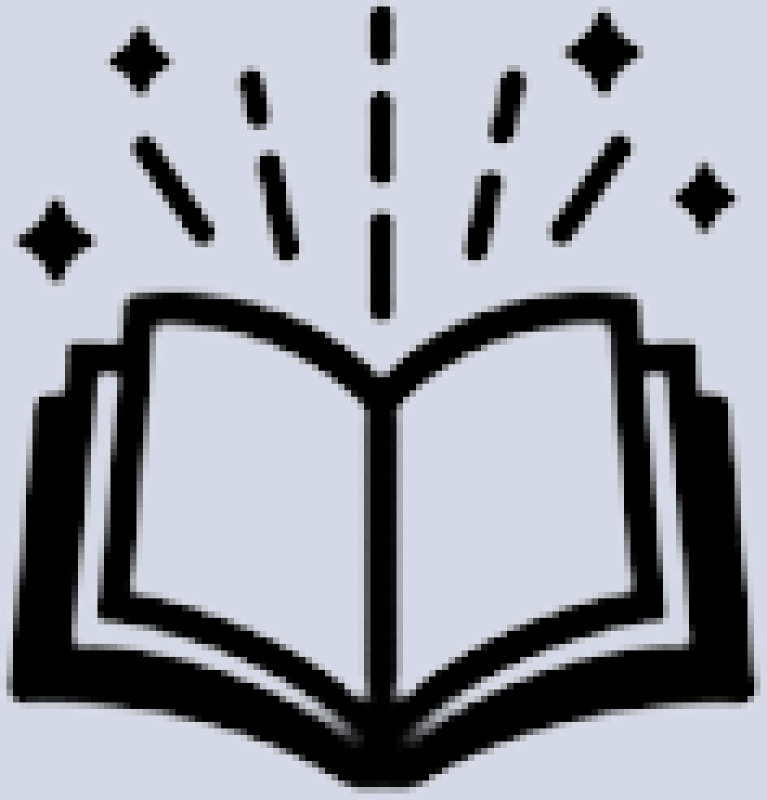
Adapting to similar infection type/setting	Cellulitis/abscess following dermatology biopsies	NHSN definitions of superficial incisional infection, deep incisional infection
	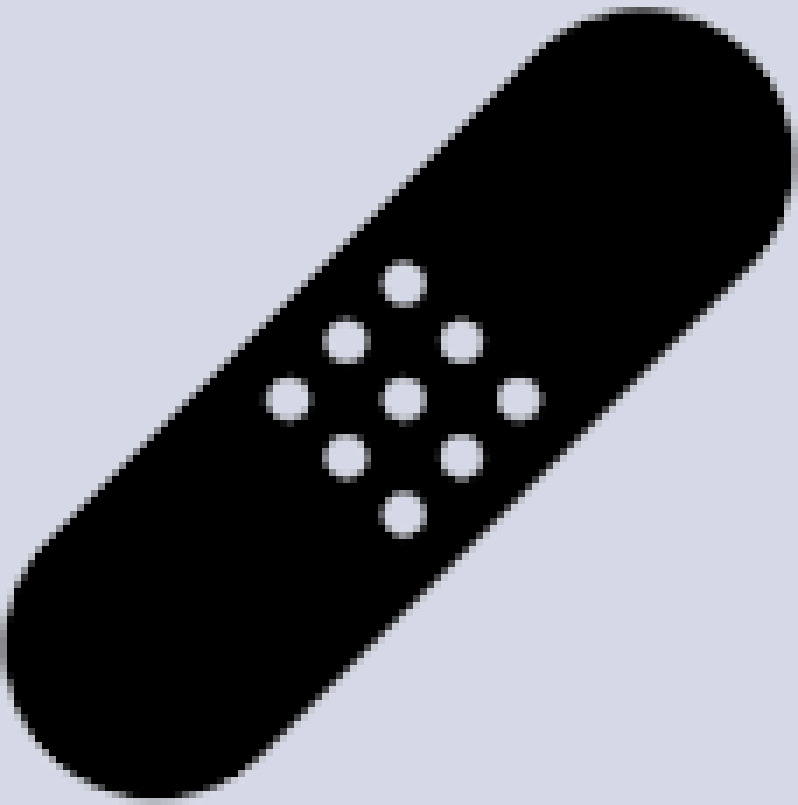	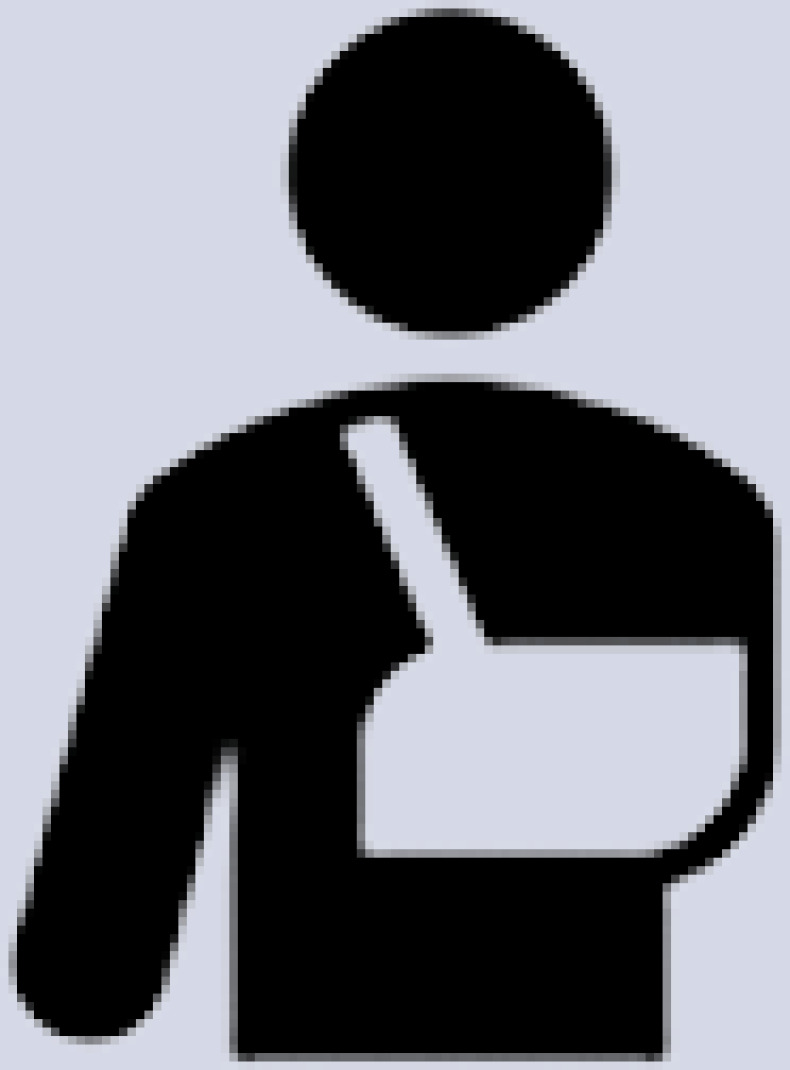
Adapting and validating clinical definition from another source	Dental infections	IDSA/SHEA, ADA clinical definitions
	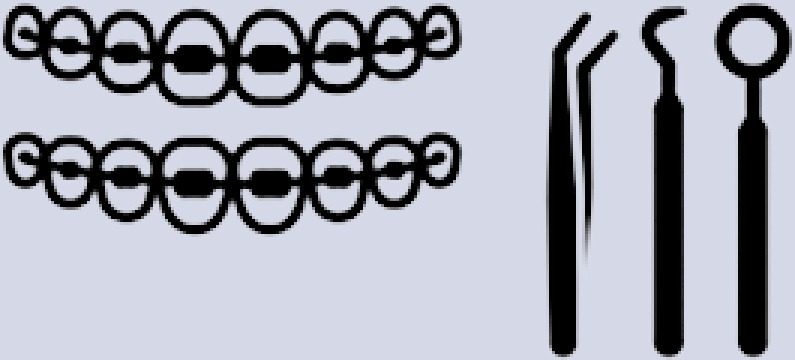 Healthcare-associated viral respiratory infections 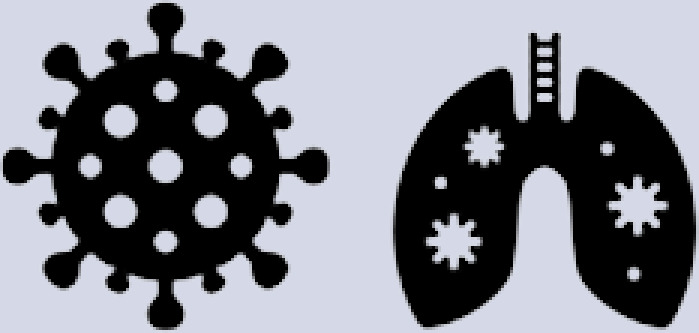	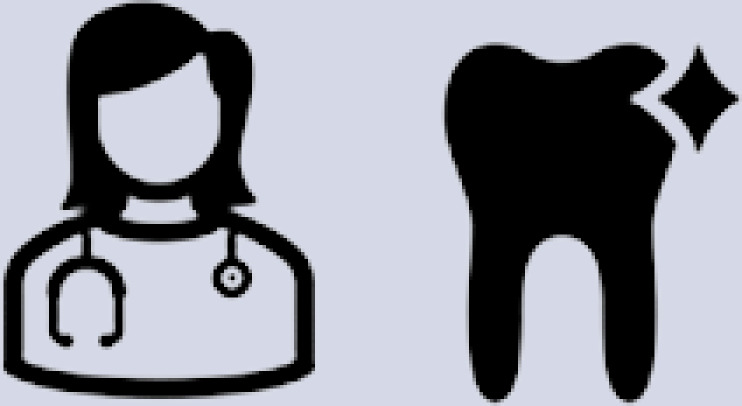 FDA drug-approval documents Objective criteria from clinical trials submissions 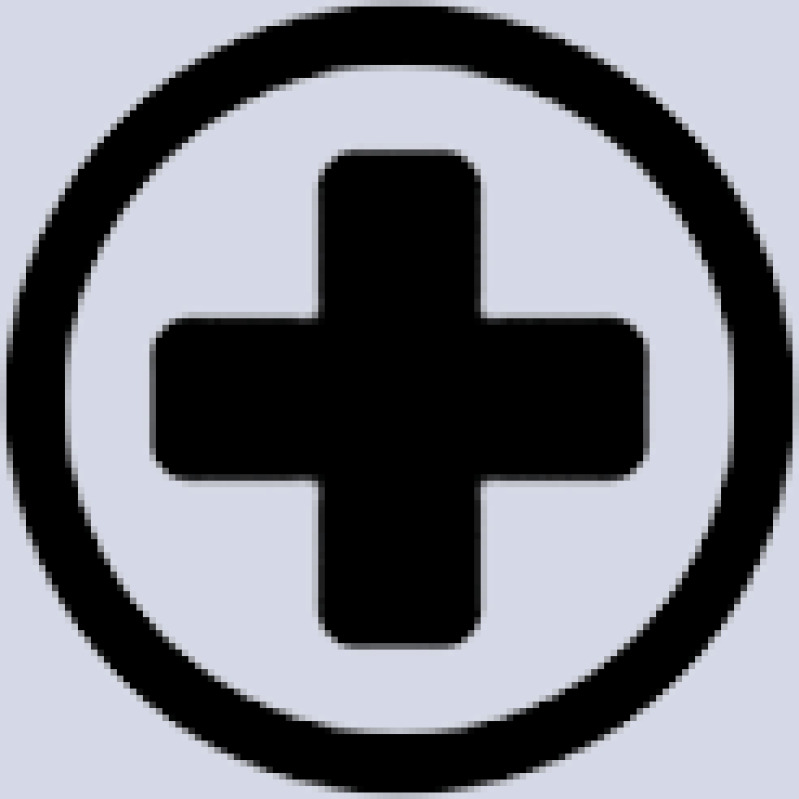
Developing definitions for emerging infections	Transfusion-related infection surveillance 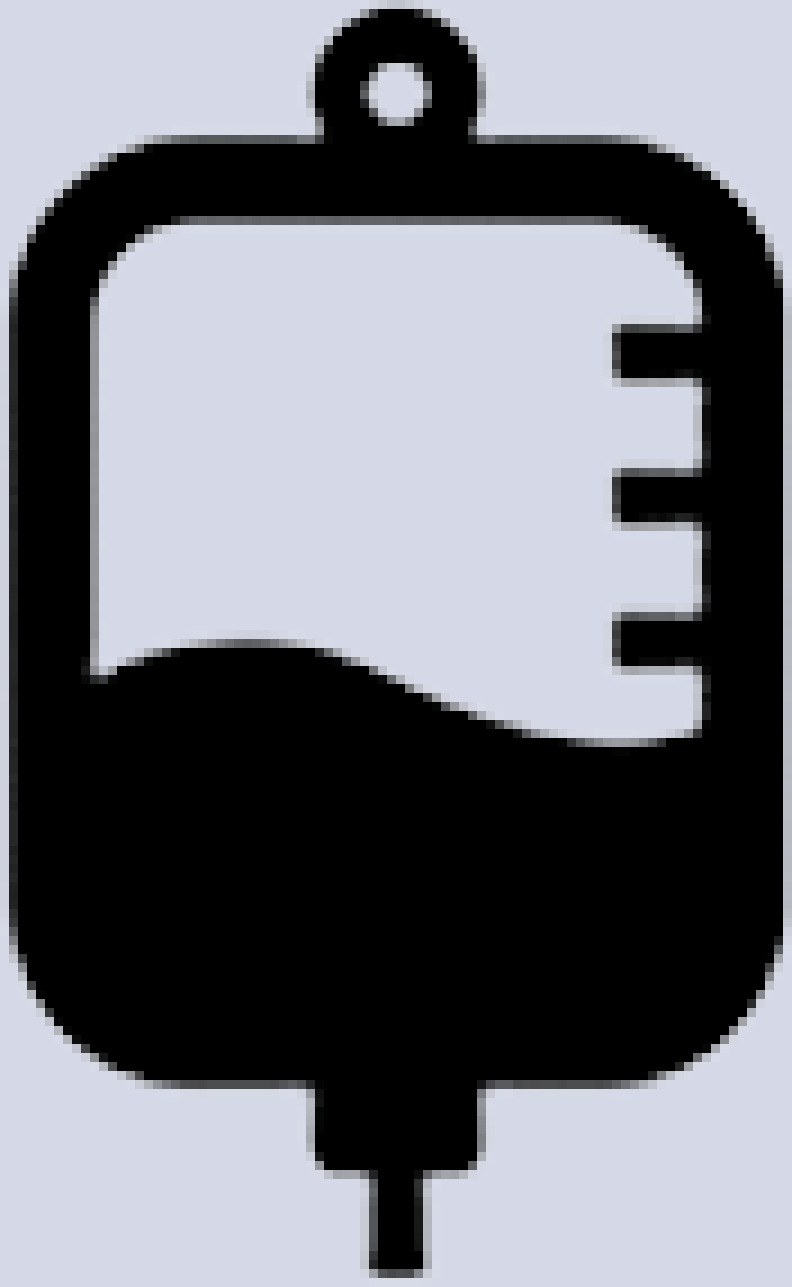 Fungal meningitis case identification 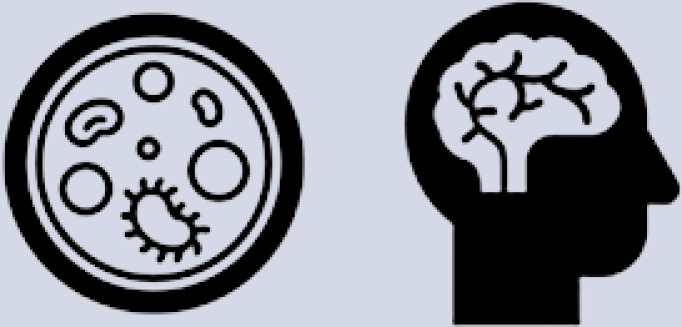	Discussion with public health and relevant experts (CDC, SHEA, IDSA) 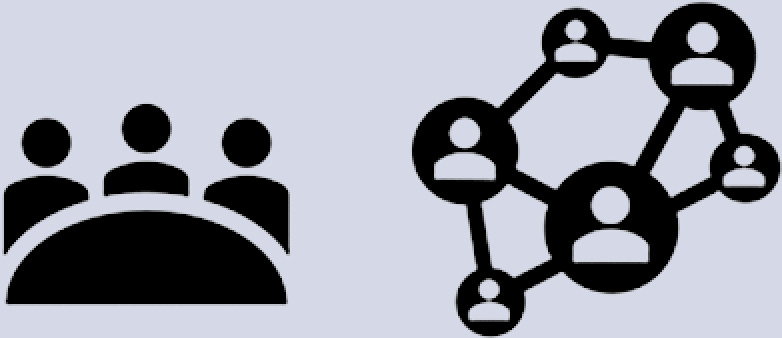

Note. NHSN, National Healthcare Safety Network; IDSA, Infectious Diseases Society of America; SHEA, Society for Healthcare Epidemiology in America; FDA, Food and Drug Administration; SHEA, Society for Healthcare Epidemiology of America; CDC, Centers for Disease Control and Prevention; ADA, American Dental Association.

Once surveillance goals are established, then the question for infection prevention departments becomes: How? The first step to addressing the question of “how” is to assess what resources and definitions are already existing that can be adapted or expanded upon to guide surveillance activities. To this end, the first question that must be addressed is this: Can I identify an operational surveillance definition developed by NHSN for the infection I am trying to measure?

**Fig. 1.**
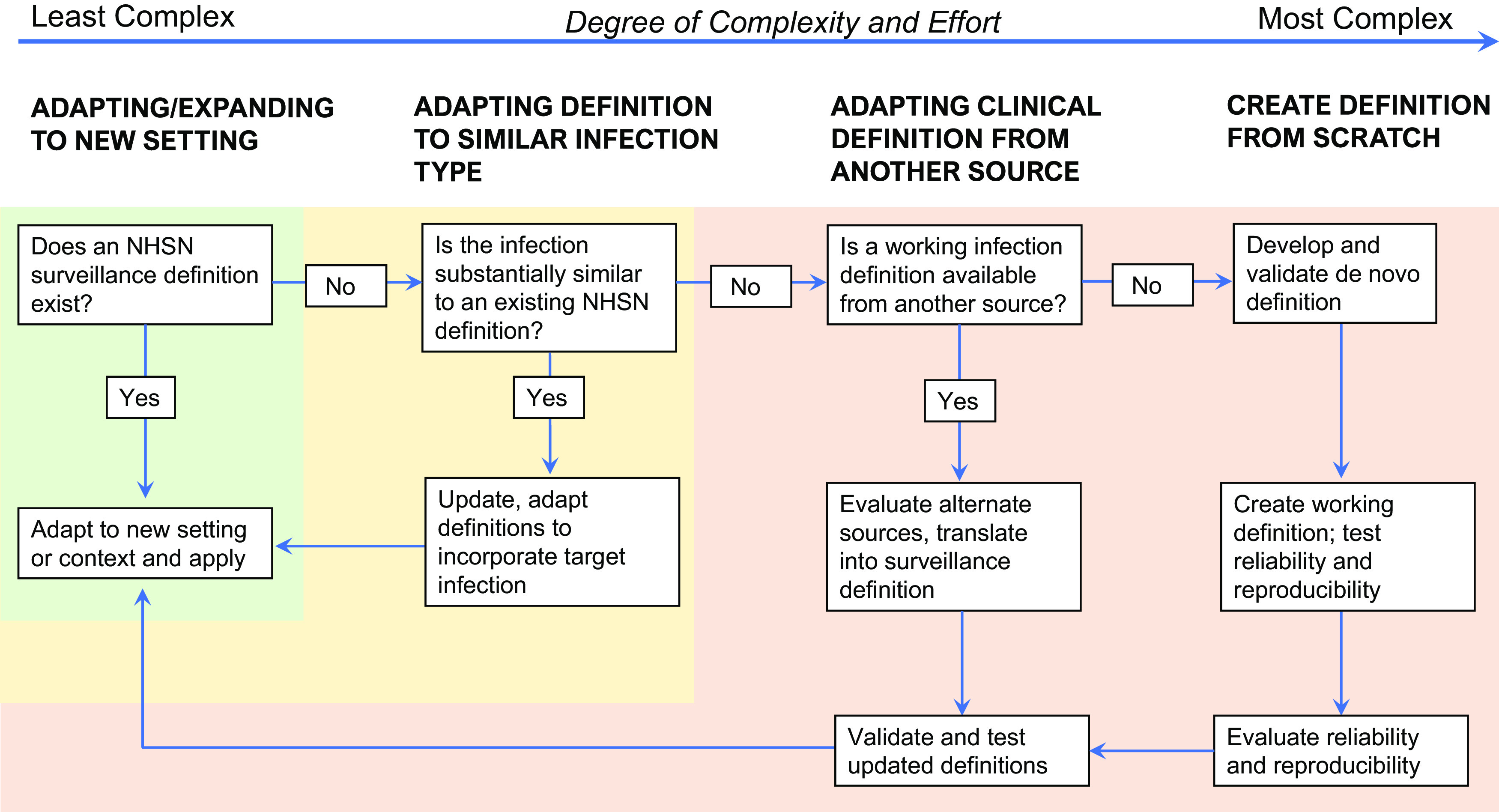
Process map for creating and expanding infection detection.

In the most straightforward circumstances, an operational surveillance definition exists, and the next steps are reasonably well-defined. Simply apply the definition to the new setting of care, perhaps with minor modification to adapt to the new context. An example of this expansion process is the application of SSI definitions to a procedure that is not included in the NHSN list of surgical procedures, such as orthopedic arthroscopies (Fig. 1). A slightly more complex but still relatively straightforward undertaking is to adapt existing definitions to infections that are functionally similar to infections for which a definition already exists; postdermatology biopsies are an example that falls into this category.

If validated NHSN definitions are not available, creating new surveillance definitions tools becomes substantially more complicated. Before embarking upon such efforts, strong consideration should be given to the face validity of measurement tools and definitions. However, if no NHSN or similar validated surveillance definitions can be adapted, then the next question to ask is this: Is there a working infection definition available from another source that can be reasonably applied to the healthcare-associated infection that I am trying to measure? A useful source of objective clinical infection definitions that have been applied for other purposes can be the US Federal Drug Administration (FDA) and clinical trials documents and registrations. For example, the clinical trial of ceftaroline for the management of pneumonia included specific inclusion criteria and outcomes assessments that could be adapted and converted into surveillance definitions.^7^ Similarly, trials of other clinical therapeutics include definitions of skin and soft-tissue infections, bone infections, and others. Because these definitions are designed for regulatory approval and interfacility agreement is critical, they generally form a reasonable starting point on which to base objective infection surveillance definitions. If FDA definitions are not available, then the next-best alternatives are definitions adapted and extracted from clinical guidelines or endorsed by professional societies, such as the Infectious Diseases Society of America guidelines for the management of MRSA infections^8^ or the American Heart Association infectious endocarditis management guidelines.^9^ Prior outbreak investigations, for example those published in academic journals or by the CDC, can also be a useful source of infection definitions. After existing definitions are translated into a surveillance definition, manual review and validation of cases to determine interrater reliability and reproducibility is necessary before moving forward with surveillance activities.

If none of this information is available, then de novo definitions must be created and validated. This process should not be embarked upon without considerable attention to need, resources, complexity, and expertise. However, recognizing that this process is complex and not ideal, it may be necessary in situations in which cases must be identified and there are no available surveillance definitions. Some examples of events that might necessitate development of entirely new definitions include surveillance for transfusion-mediated transmission by Babesia^10^ or trypanosomes^11^ and invasive fungal infections related to medication contamination, as occurred during the New England Compounding Center (NECC) outbreak.^12–14^ The latter example was particularly complicated. Because the clinical syndromes of infected patients were highly variable, infections depended upon site of exposure and included meningitis and other manifestations, such as joint infections. Further, infections did not occur within a single healthcare facility. The FDA protocol templates framework could be used as a general model and starting point for outlining and defining key features, such as study period, key end points, and exclusions.^15^

If de novo definitions are the only option, we strongly encourage gathering input from experts in the field before implementing surveillance using these definitions and validating them on a sample of true positive and true negative cases (classified after manual review), to ensure that the newly created definitions are objective and reproducible. During the validation phase, multiple blinded reviewers should apply the same definitions to the same sample of cases, and a minimum level of agreement (typically at least 80%) should be achieved before moving forward.

### Automating infection surveillance

Once a reliable, reproducible working infection surveillance definition has been developed, the next step is to establish what types of data are needed to inform automated or semiautomated infection surveillance algorithms, where these data elements are stored, and how the automated process will be applied. The identification of data elements used in automated detection tools and their location in the in the EHR is a critical step for converting infection surveillance definitions into automated infection detection tools. Data mapping, in which charts are manually reviewed and location of electronic elements is identified, can be an important part of this process, particularly if unstructured data elements are a planned element of the automated tools. For example, if a key data element is only available in clinical notes but is always contained within a specific note type or documented by a particular provider type, this information can be used to direct the automated query and the accuracy and precision of electronic data searches.

Given the denominator size, active infection surveillance processes are generally not feasible and passive surveillance processes are generally not accurate. Thus, practically, unless an infection can be defined using microbiology results only, expanded surveillance is likely to be semiautomated or ‘trigger-based,’ rather than ‘fully automated.’ In trigger-based surveillance tools, cases are screened using automated methods applied to EHRs, often using structured data elements (eg, microbiology results, emergency department visits and readmissions, administrative codes) for flagging. Cases that meet certain criteria then undergo manual chart review, typically by an Infection Preventionist, to confirm the presence of an infection. Typical triggers include (1) adverse event monitoring, as in the Institute for Healthcare Improvement Global Trigger tool,^16^ which uses events, such as emergency room visits and hospitalizations to guide adverse event detection; (2) monitoring for specific events, such as microbiology results or orders; and (3) mapping clinical treatment pathways and then identifying “roadmaps” for the clinical management of the adverse event in question and then each of the steps in the clinical management pathway in the EHR.^17^ The challenge with the intuitively appealing clinical-pathways approach is that without additional refinement, there is often a “signal to noise” problem. For example, antimicrobial use is a theoretically attractive marker for treatment and management of an infection, and therefore for HAI detection following a procedure. The challenge from a surveillance perspective is that antimicrobial use is so common that surveilling for new antimicrobial prescriptions does not distinguish between those with HAIs and those receiving antibiotics for other reasons. For example, >40% of patients undergoing cardiovascular implantable electronic device (CIED) implantation procedures receive antibiotics during the 90-day period following placement, yet the true incidence of procedure-related cardiac device infection is closer to 1.5%.^17,18^ Therefore, using antimicrobial prescriptions alone does not have sufficient specificity to reliably measure CIED infections. Similar challenges using antimicrobial prescriptions as a surveillance tool have also been described for other SSIs.^19,20^

### Unstructured data: The next frontier?

Assuming that a trigger-based process is used, the first step in the process is to determine which data elements, if any, are available as structured data elements. As discussed in detail in the first review in this series,^2^ these are generally the most amenable to automation. If structured data elements are not available or are not granular enough on their own to direct the surveillance process, then integrating unstructured data can be considered to further refine and improve the process of case ascertainment.

Although structured data elements are attractive for automated surveillance tools, a significant portion of EHR data is found in the form of unstructured clinical documentation, such as clinician progress and procedure notes, discharge summaries, and imaging and pathology reports. These unstructured data provide rich but largely untapped information for supporting surveillance activities and quality and process improvements, and, in theory they could be used to support a ‘fully automated’ infection detection system. For example, they could be used to measure data elements, such imaging and pathology reports, that are not generally currently available as structured data elements. Technological advances in data science, including natural language processing (NLP) and machine learning (ML), present a path forward for leveraging these sort of data elements to enhance and expand surveillance activities. However, significant barriers remain that prevent ‘fully’ automating infection detection activities, even if more advanced informatics methods are used.

Unstructured data can be queried in a variety of ways. In the simplest and most straightforward data extraction process, keyword searches identify the presence (or absence) of typical clinical phrases can be applied. For example, if the goal was to identify CIED infections, examples of key words might include searches such as “endocarditis,” or “cardiac device infection” or “pacemaker infection.” In theory, targeted keyword searches directly measure clinical diagnoses and can be used to flag relevant cases for manual chart review.

In practice, however, screening clinical notes is far more complicated than a simple keyword search. Clinical documentation patterns are often complex, with a high use of negation^21–23^ (eg, ‘endocarditis not suspected’) and duplication (eg, historical endocarditis diagnosis carried forward into the problem list of current notes).^24^ Moreover, these patterns are highly variable between and within clinicians, facilities, and regions. The variability among notes can be reduced with specific note templates or specific note titles that “direct” the clinical documentation process. However, even with these filters, challenges remain regarding clinical documentation that is repeatedly carried forward in the EHR. Highlighting the scope of the challenge, these documentation patterns can even be present in discharge instructions (eg, “in the event of a fever”). Without careful consideration, such data elements could be erroneously flagged as new events. Mapping data elements before operationalizing automated measurement tools can help to alleviate some of these challenges, for example, by directing text note searches to only certain note types, by including a flag for documentation of a specific key word prior to the exposure in question, by using algorithms to identify negation phrases, or by excluding notes that occur within certain time windows and thus are unlikely to represent “true infections.”

### Putting the pieces together: Automated infection identification tools

Once developed, key word searches can be used to flag cases for review or can be combined with structured data as part of an infection detection algorithm. Combining structured and unstructured data can improve the positive predictive value of automated infection detection tools because patients with “true” infections rarely have just 1 identifier but rather have multiple identifiers (eg, clinical documentation AND antimicrobial prescription AND microbiology result or order).^25^ In the development process, different infection flags can be weighted differently to identify cases with a high probability that an adverse event occurred to direct the manual review process. This process of combining multiple different flags and restricting a search based on probability cutoffs can greatly reduce, but not eliminate, the need for manual review. However, the development and validation processes can be time-consuming and requires substantial informatics and data science support in addition to upfront manual review to establish true-positive and true-negative cases.

### Advanced informatics strategies

Beyond simple text-note searches, additional detection strategies include more advanced ML approaches and NLP. To our knowledge, neither is yet in operational, near–real-time use to support infection prevention surveillance, but there are promising retrospective studies in this area. Real-time, clinical application of these technologies in other areas is increasing, and the integration of these more advanced analytic methods into infection surveillance is likely in the near future.^26–29^ Artificial intelligence has already been applied to improve the interpretation of imaging findings, such as mammography, and it offers promise for some of the challenges of infection detection, particularly for unusual events or events with variable presentations and syndromes, as was the case in the NECC outbreak.^28^ Questions remain about operationalizing an approach that requires substantial commitment of resources, including complex programming, model maintenance, and potentially frequent updating, in order to accurately measure and detect infections longitudinally.

## Barriers to automating systems: Intra- and interfacility reliability and reproducibility and missing data

Once automated infection surveillance tools are developed, challenges remain in the conversion of retrospectively created tools for operational, near–real-time use. Converting retrospective algorithms into real-time quality-measurement tools is not straightforward due to a variety of factors, including incomplete data capture until the end of the surveillance period (or later), facility variation in documentation, treatment, and diagnostic practices, and the specifics of each EHR installation.^30^ Missing data, for a variety of reasons, is a perennial problem with any system that relies on the EHR; even the best electronic algorithm cannot “see” what is not collected.

One of the aims of automated infection surveillance is to improve accuracy and comparability across different medical centers and systems. However, the accuracy and predictive value of ML and NLP approaches are often highly specific to an individual institution, region, or EHR. Staff turnover, changes in electronic templates, new evidence that leads to practice change, or even simple changes in documentation can affect accuracy. Thus, these methods require continuous monitoring and updates to retain predictive value. Although advanced informatics approaches are intriguing, more data are needed to fully elucidate their real-world applicability and translation into clinical practice. In addition, given the substantial informatics support required to achieve these goals, the long-term cost and time savings needs to be weighed against the upfront and ongoing programming and informatics costs.

In conclusion, rich and complex EHR data can be used to expand of infection surveillance to areas with limited infection prevention support but require substantial upfront IPC effort and multidisciplinary support. Much of the EHR is in the form of unstructured data elements, such as clinical notes. Clinical documentation contains rich information, but substantial challenges remain in applying electronic data extraction tools to these elements of the EHR to support ‘fully’ automated infection detection. The impact of missing data also warrants consideration.

Given the rarity of events and the reality that EHR-based definitions that utilize diagnostic codes, laboratory results, and other structured data elements will never be 100% accurate for measuring adverse events. Thus, some component of manual chart review will be necessary to support expansion of infection surveillance activities. The reality that IPC resources will be needed to support these activities should be considered upfront and resourced appropriately.
